# Comment on ‘Causal linkage of tobacco smoking with ageing: Mendelian randomization analysis towards telomere attrition and sarcopenia’ by Park et al.

**DOI:** 10.1002/jcsm.13279

**Published:** 2023-06-14

**Authors:** Mingchong Liu, Chensong Yang, Yutao Pan, Guixin Sun

**Affiliations:** ^1^ Department of Traumatic Surgery, School of Medicine, Shanghai East Hospital Tongji University Shanghai China

As we all know, it has been proven that tobacco smoking is associated with many diseases, including sarcopenia.^1^ However, tobacco smoking as a lifestyle always affects our bodies for a quite long time, which is a great challenge for researchers to conduct a randomized controlled trial to identify the causal roles of tobacco smoking in diseases. We therefore read the recent paper by Park et al. This is a well‐designed Mendelian randomization (MR) study, using genome‐wide association studies (GWASs), which may prove the evidence of causal associations of tobacco smoking with telomere attrition and sarcopenia. These findings suggested that ever being a regular smoker in life (smoking initiation) was causally associated with shorter leucocyte telomere length (LTL), lower appendicular lean mass index (ALM), slower walking pace, and lower time spent on moderate‐to‐vigorous physical activity (MVPA).^2^

However, in this study, the high sample overlapping rate in the two‐sample mendelian randomization raised concern about the conclusion: the data sources in the study were from UK Biobank (*N* = 337 138, for aging and sarcopenia) and a GWAS meta‐analysis study named GSCAN (*N* = 1.2 million, for tobacco smoking).^3^ We carefully read the raw study of the GSCAN, and unfortunately, in the 1.2 million samples, 383 613 were from UK Biobank. According to the calculation methods for the maximum estimated value for sample overlapping rate, the cohort of aging and sarcopenia (337 138 samples) may be fully overlapped with samples for smoking (383 613 samples), which means the maximum estimated sample overlapping rate might be 100%. It was the violation of the essential assumptions of two‐sample MR. The bias caused by sample overlapping should not be ignored.^4^

Interestingly, the raw data provided by GSCAN contains a dataset without UK Biobank cohorts (https://conservancy.umn.edu/handle/11299/201564). Therefore, using the GSCAN data without UK Biobank, we tried to re‐perform the MR study by Park et al. Briefly, the data including 848 460 individuals for exposure (tobacco smoking) were from the GSCAN data without UK Biobank individuals. For outcomes, similar to Park's study, we used the summary GWAS data of the UK Biobank from the IEU database.^5^ Except for handgrip strength, the phenotypes of other outcomes were as same as the previous study: including LTL (*N* = 472 174, datasets ID: ieu‐b‐4879), adjusted appendicular lean mass (*N* = 450 243, datasets ID: GCST90000025), walking pace (*N* = 459 915, datasets ID: ukb‐b‐4711), moderate to vigorous physical activity (*N* = 377 234, datasets ID: GCST006097). In the study by Park et al., handgrip strength was defined as the average value of two hands. Because we did not have access to the detailed UK Biobank data, our study's phenotypes of handgrip strength were divided into the right hand (*N* = 461 089, datasets ID: ukb‐b‐10215) and left hand (*N* = 461 026, datasets ID: ukb‐b‐7478). In the GWAS of GSCAN data without UK Biobank, only eight SNPs associated with Smoking Initiation were identified at the significance level of *P* values <5E‐8. Therefore, another analysis at the significance level of *P* values <5E‐6 was also conducted. To satisfy the three core consumptions of MR, five strict SNPs filtration steps were set: step 1, clumping the SNPs (linkage disequilibrium (LD) *r* ^2^ > 0.01, kb = 500) 30643251; step 2, excluding the SNPs associated with confounders; step 3, excluding the SNPs in the harmonization procedures; step 4, excluding the SNPs associated with outcomes; step 5, excluding the SNPs with potential pleiotropy by MR‐PRESSO. The main MR analyses were performed using random‐effects inverse‐variance weighted (IVW) analysis, MR‐Egger regression, and weighted median test.

In our analysis, only the causal associations of smoking with LTL and walking pace were proved (Figure 1). Being a regular smoker may causally associate with lower LTL (IVW, *P* = 0.045; Weighted median, *P* = 0.008) and slower walking pace (IVW, *P* = 0.043; Weighted median, *P* = 0.017). However, the other associations found in the study by Park et al., including smoking with ALM and MVPA, were not proved. As for handgrip strength, both studies provided no significant causal estimates between smoking and handgrip strength. Moreover, no significant pleiotropic effect was founded in the main MR analysis (all MR‐Egger intercept *P* < 0.05), which supported the main estimates in our study.

**Figure 1:**
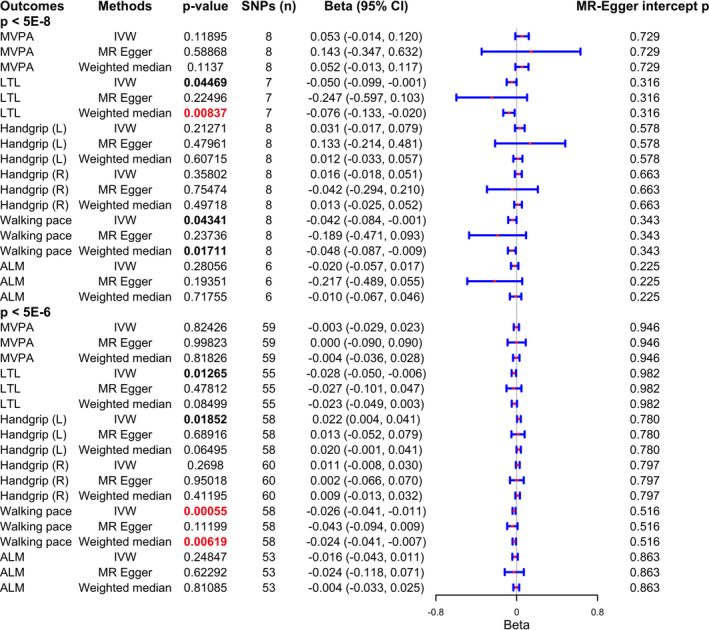
Results of Mendelian randomization in our study.

In conclusion, based on our study and previous study, we believe the causal relationships between smoking and LTL and walking pace could be proven. The causal role of smoking in ALM and MVPA may need further discussion. Moreover, we tend to believe that no causal relationship between smoking and handgrip strength exists.

In addition, according to the revised sarcopenia definition from the European Working Group on Sarcopenia in Older People (EWGSOP2), low muscle strength is the most reliable measure of sarcopenia, and sarcopenia could be confirmed by low muscle quantity or quality, while low physical performance is considered to measure the severity of sarcopenia.^6^ Considering both our and previous studies, in the sarcopenia traits, only the causal relationship between sarcopenia and the walking pace was proved, while no significant and solid evidence for muscle strength and quantity was provided. The causal effect of tobacco smoking on sarcopenia needs further studies.

## Conflict of interest

The authors declare that they have no competing interests.

## Funding

National Natural Science Foundation of China (Grant/Award Number:81971169) Leading Talents Training Program of Pudong New Area Health Commission (Grant ID: PWR 12020‐06).
